# Spatial Transcriptomics in Chronic Rhinosinusitis: Deciphering Tissue Architecture, Immune Niches, and Precision Therapeutics

**DOI:** 10.3390/ijms27219510

**Published:** 2026-10-24

**Authors:** Subhajit Ghosh, Inamul Hasan Madar, Koushik Bardhan, Punitha Muruganantham

**Affiliations:** 1 Centre for Integrative Omics Data Science (CIODS), Yenepoya (Deemed to be University), Mangalore 575018, Karnataka, India; subhajit.ghosh.academic@gmail.com (S.G.);; 2 Centre for Health Informatics and Evidence Based Medicine (CHIEM), Yenepoya (Deemed to be University), Mangalore 575018, Karnataka, India; 3 Multiomics and Precision Medicine Lab (MPML), Yenepoya Research Centre (YRC), Yenepoya (Deemed to be University), Mangalore 575018, Karnataka, India; 4 Department of Bioinformatics, University of North Bengal, Siliguri 734013, West Bengal, India

**Keywords:** chronic rhinosinusitis, chronic rhinosinusitis with nasal polyps, spatial transcriptomics, single-cell RNA sequencing, immune niche, tissue remodelling, evidence grading, precision medicine

## Abstract

Chronic rhinosinusitis (CRS) is a heterogeneous inflammatory disorder in which epithelial injury, immune activation, and stromal remodelling are organised into local tissue niches. Bulk and single-cell RNA sequencing have defined important cell states, but dissociation removes the anatomical relationships needed to determine where those states coexist. Spatial transcriptomics (ST) addresses this gap by mapping RNA, and in some platforms, protein, onto intact sinonasal tissue. This review provides an evidence-graded synthesis organised around a single translational chain: spatial niche to molecular signature to plausible biological mechanism to CRS phenotype/endotype to clinical implication to therapeutic relevance to validation status. Direct CRS ST evidence is separated from single-cell RNA sequencing support, computational prediction, protein or in situ evidence, experimental perturbation, clinical validation, and extrapolation from other inflammatory diseases. Current CRS studies support spatial compartmentalisation of eosinophil-rich epithelial lesions, ALOX15-positive macrophage programmes, GZMK-positive CD8 T-cell-fibroblast neighbourhoods, and treatment-associated epithelial and stromal changes. However, most ligand-receptor calls remain predictive, and spatial association alone does not establish functional causality. ST is therefore best viewed as a discovery and mechanistic-prioritisation technology whose clinical promises—biomarker selection, biologic matching, and response monitoring—require independent cohorts, locked assays, patient-level reproducibility, and prospective outcome studies. We compare study designs and platforms, examine statistical issues including spatial autocorrelation and pseudoreplication, and propose a cautious translational framework in which orthogonal and clinical validation convert spatial observations into actionable evidence.

## 1. Introduction

Chronic rhinosinusitis (CRS) is an inflammatory disorder of the sinonasal mucosa lasting at least 12 weeks and affecting approximately 10-12% of adults worldwide [1]. CRS is the umbrella diagnosis; chronic rhinosinusitis with nasal polyps (CRSwNP) and chronic rhinosinusitis without nasal polyps (CRSsNP) are clinical phenotypes. Eosinophilic CRSwNP (eCRSwNP) denotes a tissue-inflammatory subgroup defined by eosinophil-rich pathology. Thresholds vary among studies: reported definitions include >20% eosinophils among inflammatory cells, ≥70 eosinophils per high-power field, >55 per high-power field, and ≥10 per high-power field [2]. Cross-study comparison therefore requires reporting the threshold, field size or magnification, counting method, and sampling site; where available, study-specific thresholds in direct spatial studies are provided in Supplementary Tables S1 and S2. Type 1, type 2, and type 3 designate immune endotypes dominated respectively by interferon-associated, IL-4/IL-5/IL-13-associated, and IL-17-associated programmes. Phenotype and endotype are not interchangeable: CRSwNP is frequently, but not invariably, type 2, and mixed programmes can coexist within one patient or specimen [3]. Another recent clinical overview supports this epidemiological and management context [4].

Bulk transcriptomics established dysregulation of type 2 cytokines, interferons, IL-17, transforming growth factor-β (TGF-β), and epithelial alarmins but average signals across heterogeneous tissue. Single-cell RNA sequencing (scRNA-seq) resolves epithelial, immune, endothelial, and mesenchymal cell states, yet dissociation removes their native neighbourhoods. ST retains tissue coordinates and can therefore connect cell identity with local epithelial damage, immune recruitment, extracellular-matrix (ECM) remodelling, and vascular change. Its core contribution is spatial association: a transcript-rich region or neighbouring cell pair can nominate a mechanism but does not alone demonstrate secretion, receptor engagement, or causality. Earlier bulk transcriptomics and later multi-omic single-cell works further document molecular heterogeneity across CRS phenotypes [5,6].

The direct CRS spatial literature remains compact. Liu et al. combined digital spatial transcriptomics, scRNA-seq, and functional co-culture to link ALOX15-positive macrophage programmes with eosinophilic inflammation and epithelial remodelling [7]. Guo et al. used Visium, Visium HD, immune-receptor sequencing, multiplex imaging, and fibroblast perturbation to define GZMK-positive CD8 T-cell-fibroblast neighbourhoods; its ligand-receptor maps are predictive, whereas the fibroblast response to GZMK received functional support [8]. Liao et al. integrated single-cell discovery with GeoMx Digital Spatial Profiling (DSP) in an independent cohort of more than 100 participants, identifying macrophage, T-cell, mast-cell, basal-cell, and tuft-cell programmes across CRSwNP, CRSsNP, and healthy tissue [9].

Moreover, spatial profiling has also begun to address treatment response. West et al. analysed paired nasal-polyp biopsies from 20 participants before and during mepolizumab treatment and observed region-specific RNA and protein changes involving immunity, TGF-β-associated stromal programmes, epithelial barrier function, and ciliogenesis [10]. This repeated-measures design is translationally informative, but it does not establish that any baseline spatial signature selects responders. Related scRNA-seq, histology, immunostaining, and organotypic studies, including epithelial inflammatory memory [11], provide mechanistic support.

Together, the data support a model in which CRS arises from interacting epithelial, immune, and stromal niches rather than uniform mucosal inflammation. The key analytical sequence is where a molecular programme occurs, which cells contribute to it, what mechanism it may represent, how it relates to phenotype or endotype, and what level of validation supports a clinical inference. This sequence allows spatial maps to generate testable biomarkers and therapeutic hypotheses while preventing overinterpretation of co-localisation or computational communication scores.

The distinctive contribution of this review is therefore an evidence-graded, translational synthesis of CRS spatial biology. Rather than listing platforms and studies sequentially, it connects spatial niches to molecular signatures, mechanisms, disease phenotypes/endotypes, potential clinical applications, and the validation steps still required. Throughout, therapeutic and precision-medicine claims are framed as current evidence, emerging application, or future possibility.

### 1.1. Scope, Terminology, and Evidence Framework

Evidence descriptors used throughout are as follows: direct CRS ST evidence; scRNA-seq-supported evidence; computational prediction; protein or in situ evidence; experimental/functional validation; clinical validation; and extrapolation from other inflammatory diseases. These categories describe what was measured and should not be collapsed into a single level of certainty.

A spatially enriched gene or adjacent cell pair is described as spatially demonstrated only when measured in a CRS spatial dataset. Ligand-receptor analyses are described as computationally predicted unless the pair has been confirmed by protein localisation, pathway activation, or perturbation. Clinical validation is reserved for prospectively or independently demonstrated association with patient outcomes or treatment utility.

### 1.2. Literature Search Strategy

This article is a narrative review, not a systematic review or meta-analysis. We searched PubMed/MEDLINE, Scopus, and Web of Science Core Collection from database inception through 12 July 2026. The core search combined CRS terms (“chronic rhinosinusitis”, “nasal polyp*”, and “sinonasal”) with spatial-omics terms (“spatial transcriptom*”, “spatial omics”, “digital spatial profiling”, “Visium”, “Xenium”, “CosMx”, “MERFISH”, and “seqFISH”). Searches were supplemented with terms for “single-cell RNA sequencing”, “endotype”, “eosinophil*”, “macrophage*”, “fibroblast*”, “biologic*”, and “precision medicine” and by screening cited and citing records. We included peer-reviewed English-language original studies in human CRS that used spatial molecular profiling or provided directly relevant single-cell, in situ, protein, functional, clinical, or methodologic support; representative reviews were used for field-level context. We excluded duplicate records, conference abstracts without full data, reports with no direct CRS/sinonasal relevance, and non-human studies unless they provided clearly identified mechanistic support. 

## 2. Spatial Transcriptomic Technologies for Sinonasal Research

**Figure 1:**
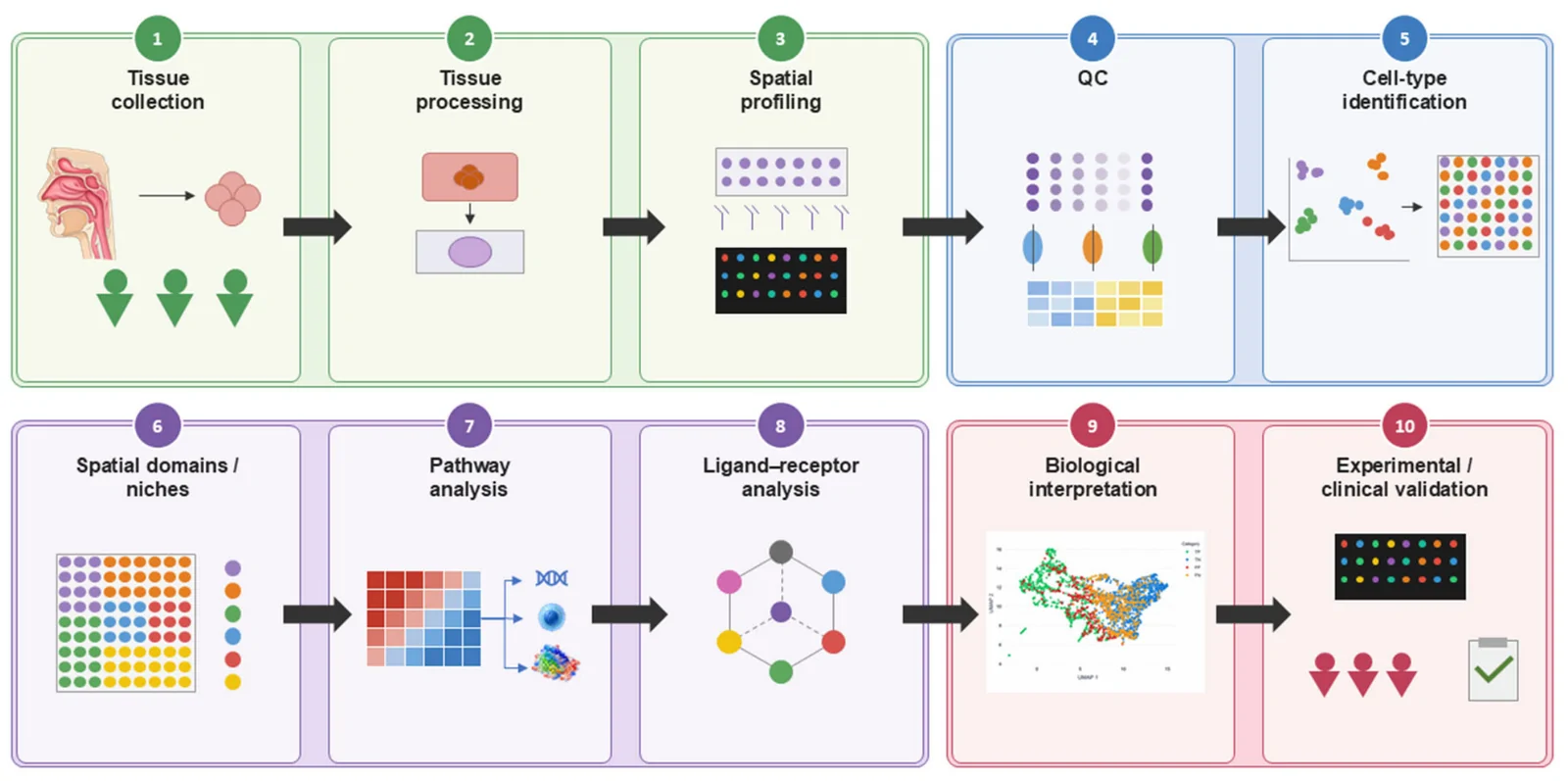
Complete ST workflow for CRS. Quality control and patient-level statistical design span the analytical pipeline. Ligand-receptor analysis is explicitly treated as predictive until supported by protein localisation, perturbation, or clinical evidence.

Spatial technologies differ in transcript coverage, resolution, tissue compatibility, throughput, and computational burden. Platform choice should follow the biological question: unbiased discovery, high-resolution localisation, protein confirmation, or targeted clinical validation. In CRS, archived formalin-fixed paraffin-embedded (FFPE) surgical material favours robust ROI- or probe-based workflows, whereas fresh-frozen tissue can support broader transcriptome discovery. Table 1 compares the direct CRS spatial studies; Figure 1 shows an evidence-aware workflow from tissue collection to experimental and clinical validation.

### 2.1. Evolution of Spatial Transcriptomics

Early sequencing-based ST used spatially barcoded arrays to capture polyadenylated RNA from intact sections, providing broad transcript coverage at multicellular spot resolution. Higher-density arrays and imaging-based chemistries now approach cellular or subcellular localisation, whereas ROI-based platforms trade unbiased whole-slide mapping for robust profiling of selected histological compartments. Imaging platforms can localise individual transcripts but generally require a predefined panel; sequencing platforms offer broader discovery but demand deeper sequencing and deconvolution. Multi-omic extensions add protein, chromatin, or metabolite measurements, yet alignment and modality-specific sensitivity remain important sources of uncertainty.

### 2.2. Major Spatial Transcriptomic Platforms

#### 2.2.1. 10x Genomics Visium

10x Genomics Visium captures spatially barcoded RNA over a defined tissue area and aligns sequencing output with histology [13]. Standard Visium uses 55 μm capture spots that usually contain multiple cells; Visium HD uses a denser array of 2 × 2-μm bins that can be aggregated or segmented toward single-cell analysis. Both are sequencing-based and suitable for broad discovery, but cellular assignment depends on morphology, segmentation and/or reference-guided inference. For CRS, Visium can map unbiased transcriptome programmes across epithelium, stroma, and immune aggregates. Guo et al. applied standard Visium and Visium HD to nasal-polyp and control tissues [8]. Resolution should not be equated with biological replication: thousands of bins from one section remain observations nested within one patient.

#### 2.2.2. NanoString GeoMx Digital Spatial Profiler

GeoMx DSP quantifies RNA and/or protein in investigator-defined regions of interest (ROIs) selected using morphology markers [14]. It is especially compatible with FFPE tissue and can separately profile epithelial, stromal, immune, or vascular areas, making it attractive for archived CRS cohorts and paired treatment biopsies.

The approach has supported major CRS studies, including multi-scale endotype mapping and longitudinal mepolizumab profiling [9,10]. ROI aggregation improves sensitivity but is not inherently single-cell, and conclusions depend on how regions are selected and segmented. Because selection is morphology guided, unchosen or unexpected niches can be missed. GeoMx data should therefore be analysed at the patient level with ROIs modelled as repeated observations. However, it is important to note that many ROIs from the same participant increase spatial coverage, not the number of independent biological replicates. 

#### 2.2.3. MERFISH and seqFISH+

Multiplexed error-robust fluorescence in situ hybridisation (MERFISH) and sequential fluorescence in situ hybridisation (seqFISH+) detect predefined RNA targets through iterative imaging at cellular or subcellular resolution [15,16]. They are well suited to localising candidate epithelial states, rare immune cells and spatial gradients after discovery. Because individual molecules are imaged, these platforms reduce spot mixing and can support explicit cell-neighbourhood analysis. Their accuracy still depends on tissue autofluorescence, probe performance, image registration and cell segmentation; a segmented cell boundary is an analytical estimate rather than a direct biological measurement. No major human CRS cohort reviewed here has yet used MERFISH or seqFISH+ as its principal spatial platform. Proposed applications to IL-13-responsive epithelium, eosinophil-rich foci, or macrophage-fibroblast adjacency are therefore future validation strategies, not demonstrated CRS results. Targeted panels may miss unanticipated pathways, while repeated imaging increases acquisition time and data volume. Table 2 audits representative CRS ligand-receptor axes and identifies the source study, spatial platform, evidence type, validation method, and residual uncertainty.

#### 2.2.4. Xenium In Situ

Xenium In Situ uses iterative imaging to quantify a targeted RNA panel and assign transcripts to segmented cells. Current assays can profile thousands of genes in FFPE or frozen tissue without next-generation sequencing, offering high spatial precision for candidate validation.

In CRS, Xenium could test whether candidate epithelial progenitors, fibroblasts, eosinophils, mast cells, and lymphocytes occupy predicted neighbourhoods. This is a prospective use: the direct CRS studies summarised in Table 1 did not rely on Xenium. Performance depends on panel design, transcript abundance, morphology, and segmentation quality. Accordingly, Xenium is best framed as a targeted localisation and validation platform rather than an already established CRS clinical assay. More generally, spatial mapping in high-grade glioma illustrates how heterogeneous tissue domains can be resolved in other diseases [20]. 

#### 2.2.5. CosMx Spatial Molecular Imaging

CosMx Spatial Molecular Imaging combines highly multiplexed RNA imaging with optional protein measurement at single-cell/subcellular scale [21]. It can bridge transcript localisation and protein context without next-generation sequencing, although panel content and throughput are assay dependent. For CRS, simultaneous RNA and protein measurements could help determine whether a candidate cytokine-producing cell also contains the relevant protein and lies near a receptor-bearing target. Such concordance is stronger than RNA-only co-expression but still requires perturbation to establish functional communication.

#### 2.2.6. Emerging High-Resolution Platforms

Stereo-seq and Seq-Scope combine dense capture arrays with sequencing to achieve subcellular-scale coordinates over relatively large tissue areas [22,23]. They offer powerful unbiased discovery but require specialised laboratory workflows, deep sequencing, and substantial computation. Slide-seqV2 provides near-cellular capture through barcoded beads but has more limited clinical standardisation. Their proposed value for ciliary polarity, glandular organisation, and micro-niches in CRS remains untested in large patient cohorts. Practical platform parameters, including specimen compatibility, throughput, tissue area, sequencing burden, and discovery-versus-validation use, are compared in Table 3.

### 2.3. Computational Analysis of Spatial Transcriptomic Data

Spatial analysis should begin with a prespecified unit of inference. The patient—not the spot, bin, ROI, or segmented cell—is generally the independent biological replicate when multiple observations arise from one participant. Treating thousands of spatial observations from a few patients as independent creates pseudoreplication, underestimates variance, and inflates significance. Designs should include multiple patients per group, balanced batches, and, where feasible, multiple anatomical sites or sections nested within patients. Seurat can transfer scRNA-seq labels and integrate modalities; Squidpy constructs neighbourhood graphs and evaluates spatial autocorrelation; BayesSpace refines spot-level domains; SpaGCN combines expression, coordinates, and histology. These tools answer different questions, and their outputs should not be treated as direct measurements. Spatially variable-gene and neighbourhood tests require multiple-testing control and null models or permutations that preserve spatial structure. Patient-level pseudobulk, hierarchical or mixed-effects models, and sensitivity analyses across sections are preferable to spot-level tests that ignore within-patient correlation.

Cell2location and related deconvolution methods estimate cell abundance in mixed spots, whereas Tangram maps reference cell states to spatial coordinates. Results depend on reference-dataset composition, annotation quality, platform-specific gene detection, and model assumptions. Rare or disease-specific states missing from the reference may be misassigned. Studies should report posterior or confidence measures, test alternative references, quantify segmentation error, and distinguish observed transcripts from imputed cell identities. Batch effects and cross-platform normalisation should be assessed at the patient and assay levels rather than removed so aggressively that biological differences disappear. Artificial intelligence (AI) can assist segmentation, feature extraction, and morphology-to-expression prediction. Demonstrated performance must be separated from future potential. Models should report cohort size, data partitioning at the patient level, internal versus external validation, and calibration and performance metrics and should prevent tiles from the same slide or patient leaking across training and test sets. 

### 2.4. Current Challenges in Technology Selection

No single platform satisfies all CRS research needs. Sequencing-based assays support relatively unbiased discovery but require next-generation sequencing and may mix cells; imaging-based assays provide cellular localisation but are often targeted and computationally intensive; ROI-based platforms work well with FFPE but depend on region selection. Resolution, transcript coverage, tissue area, assay sensitivity, turnaround, cost, and available expertise must be considered together rather than ranked by nominal pixel or bin size alone.

A defensible translational design uses broad spatial discovery in a patient-diverse cohort, orthogonal localisation by RNA/protein imaging, functional perturbation of prioritised mechanisms, and independent clinical validation with prespecified outcomes. Table 3 provides an airway-mucosal reference for the resolution, capture area/throughput, sensitivity, and implementation trade-offs of major platforms [24].

#### CRS Tissue Handling and Quality Control

Sinonasal specimens require procedures that preserve a thin, fragile epithelium alongside mucus-rich luminal material and variably fibrotic stroma. At collection, the operative site, prior corticosteroid or biologic exposure, ischaemia time, orientation, fixation or freezing method, and storage conditions should be recorded. On serial sections, pathologist review should delineate intact epithelium, subepithelium/lamina propria, gland, vascular, and artifactual regions; mucus and necrotic material should not be interpreted as tissue capture area. Platform-specific quality control should include RNA integrity or DV200 where appropriate, tissue coverage and permeabilisation or probe-hybridisation performance, reads/transcripts/UMIs per capture unit, negative-control signal, segmentation plausibility, and the spatial distribution of failed regions. Pilot optimisation should precede cross-patient comparisons, and thresholds should be justified and applied consistently [25].

## 3. Decoding the Spatial Immune Landscape of Chronic Rhinosinusitis

The most consistent contribution of ST to CRS is evidence that molecular activity is compartmentalised. Direct CRS spatial studies show region-specific epithelial, immune, and stromal programmes, but the strength of evidence varies by conclusion. A mapped transcript or cell-type estimate supports spatial association; scRNA-seq strengthens cell-state interpretation; protein imaging confirms localisation; perturbation tests mechanism; and clinical studies establish relevance to outcomes.

This evidence ladder is essential when moving from niche to mechanism. Type 2, type 1, type 3, and mixed programmes may occupy different areas of the same specimen, and phenotype labels such as CRSwNP or CRSsNP do not uniquely specify the active endotype. Disease severity may therefore reflect both the magnitude and organisation of inflammation.

The following sections distinguish direct spatial findings from supporting non-spatial evidence and computational hypotheses. Table 4 relates candidate spatial endotypes to currently validated biologic mechanisms while making clear that spatial treatment-selection biomarkers have not yet been clinically validated.

### 3.1. Spatial Organization of Type 2 Inflammation

Type 2 inflammation is common in CRSwNP and is characterised by IL-4/IL-5/IL-13 activity, eosinophils, mast cells, IgE, and group 2 innate lymphoid cells (ILC2s). Direct CRS spatial studies support non-uniform epithelial and subepithelial type 2 programmes and macrophage-associated eosinophilic niches [7,9]. These data demonstrate regional association, not that every nominated ligand-receptor pair is active. Chemokines such as CCL26 and CCL13 have protein, cellular, or functional support in CRS, whereas their precise sender-receiver relationships require pair-specific validation. Orthogonal evidence includes increased thymic stromal lymphopoietin activity, type 2 mucin regulation, eosinophil biology, and serum IL-5/periostin/IL-33 associations [48,49,50,51].

### 3.2. Eosinophil Niches as Spatial Signaling Centers

Eosinophils often concentrate below damaged epithelium and within inflammatory or vascularly accessible regions rather than being evenly distributed. Spatial datasets detect eosinophil-associated transcripts and neighbouring epithelial or stromal programmes, while histology and protein studies confirm eosinophil-rich foci. Because spot-based platforms may mix several cells, cellular localisation should be verified with in situ RNA or protein imaging.

Genes encoding eosinophil granule proteins (for example CLC, EPX, PRG2, and RNASE2) and epithelial type 2 responses (including POSTN, ALOX15, and MUC5AC) can mark these regions. Co-occurrence is consistent with reciprocal epithelial-immune signalling, but RNA abundance does not establish protein release, directionality, or receptor activation. IL-33-ST2 and CCL26-CCR3 therefore receive separate evidence labels in Table 2 and Table 5. Eosinophil-epithelial and eosinophil-associated remodelling mechanisms are also supported by co-culture, mucociliary-differentiation, and intercellular adhesion molecule-1 studies [52,53,54].

Fibroblast activation and ECM deposition near eosinophil-rich regions are supported by histology and non-spatial functional studies. Computational ligand-receptor analysis can nominate eosinophil-fibroblast communication, but direct perturbation of the proposed pair in an intact human CRS niche is generally lacking. The defensible conclusion is that eosinophil location and activation state may contribute to remodelling, not that ST alone proves a causal circuit. Nasal-fibroblast experiments support roles for IL-25 and eosinophil-derived osteopontin, while a dedicated review summarizes fibroblast contributions to airway remodelling [55,56,57]. Thus, eosinophil-rich niches are useful mechanistic and biomarker candidates, but clinical utility requires reproducible sampling, quantitative cut-offs, and prospective association with recurrence or treatment response beyond conventional tissue and blood eosinophilia.

### 3.3. Macrophage Heterogeneity Beyond the M1/M2 Paradigm

Single-cell and spatial studies show that CRS macrophages occupy multiple states that are not captured by a binary M1/M2 model. ALOX15-positive and CCL13/CCL18-associated macrophage programmes are enriched in eosinophilic disease and localise near epithelial and immune compartments [7,9,31]. In Liu et al., co-culture and pathway perturbation provided functional support for macrophage-epithelial remodelling, although the complete in vivo communication network remains unresolved. SPP1 (osteopontin)-high macrophages are well described in fibrotic and inflammatory tissues, but extrapolation to CRS should be explicit. A 2026 CRS study integrated public bulk and scRNA-seq data with human tissue staining and a murine metabolic intervention and used CellChat to predict SPP1-CD44 communication (DOI: 10.1186/s12931-026-03769-1). This supports the presence and metabolic regulation of SPP1-positive macrophages; it does not provide direct CRS ST evidence or prove SPP1-CD44 signalling in human tissue. In fibrotic lung and intestinal inflammation, independent single-cell/spatial studies and a cross-disease review provide contextual support for SPP1-positive macrophage states, but these data are extrapolative for CRS [58,59,60]. Macrophage state is shaped by cytokines, hypoxia, ECM, and neighbouring cells. Spatial proximity can therefore prioritise ALOX15-, CCL13-, CCL18-, or SPP1-associated niches for perturbation. To apply the evidence framework consistently, SPP1-CD44 is treated throughout this review as an extrapolated, non-spatial CRS hypothesis rather than a direct CRS ST result.

### 3.4. Group 2 Innate Lymphoid Cells as Tissue Organizers

ILC2s respond to epithelial IL-25, IL-33, and thymic stromal lymphopoietin (TSLP) and can produce IL-5 and IL-13. CRS tissue studies and ex vivo experiments identify IL-33-responsive ILCs as a local IL-13 source in nasal polyposis [61], and ST/scRNA-seq atlases support proximity among epithelial progenitors, tuft-like cells, and type 2 populations [9]. Spot-based data may not resolve every ILC2, so the IL-33-ST2 axis is supported by protein/cellular evidence rather than claimed as a directly demonstrated CRS spatial-omics interaction. Additional CRS tissue and review evidence links IL-25-responsive epithelium and expanded ILC2 populations to CRSwNP and eCRSwNP [62,63,64,65]. ILC2s are therefore plausible organisers linking epithelial injury to eosinophilic inflammation and secretory remodelling. 

### 3.5. B Cells, Plasma Cells, and Tertiary Lymphoid Structures

Tertiary lymphoid structures (TLSs) are organised B-cell, T-cell, and plasma-cell-rich aggregates that can form in chronically inflamed nasal polyps. Histology, immunostaining, and transcriptomic studies support local CXCL13, CCL19, CCL21 associated organisation and immunoglobulin production [26,27,28]. Direct high-resolution ST characterisation across large CRS cohorts remains limited. B-cell-attracting chemokines and long-lived plasma cells provide complementary non-ST evidence for local humoral organisation [29,30]. These structures provide a plausible local source of sustained antibody and adaptive immune activity, but their presence does not prove that they cause treatment resistance or recurrence. 

### 3.6. Neutrophilic Niches in Non-Type 2 CRS

Type 1, type 3, neutrophilic, and mixed endotypes are variably represented across populations and can occur in CRSwNP or CRSsNP. Geographic differences are group-level trends rather than patient-level rules. Neutrophil-rich tissue commonly shows antimicrobial, degranulation, and epithelial-injury programmes, with S100A8, S100A9, and LCN2 supported by transcript and protein studies [32,33]. Reviews and regional cohorts document population-dependent type 2/non-type 2 balance and eosinophilic-neutrophilic overlap [34,35,36].

Single-cell analysis demonstrates heterogeneous neutrophil states in nasal polyps [37], but comprehensive CRS spatial mapping of neutrophil trajectories remains less developed than type 2 mapping. Apparent proximity to damaged epithelium is biologically plausible and can be tested with imaging, yet ST alone cannot determine whether neutrophils initiated or followed barrier injury. IL-17-associated and innate inflammatory programmes may contribute to steroid resistance, but targeted treatment in CRS remains investigational. Spatial detection of a type 3 or neutrophilic region is not presently a validated indication for a specific biologic. Prospective trials must test whether a spatial signature adds predictive value beyond phenotype, blood counts, tissue histology, and comorbidities. IL-17A has direct tissue and cellular support in CRSwNP, but its value as a spatial treatment-selection marker remains unproven [38]. Within non-type 2 CRS, ST can test whether interferon-dominant, IL-17-dominant, neutrophil-rich, and remodelling programmes coexist in a patient or occupy distinct mucosal niches. Priorities include CRSsNP and non-polypoid specimens, prospective sampling of neutrophilic disease across geographical settings, and analyses that distinguish type 1, type 3, and mixed states rather than treating all non-type 2 inflammation as one category. 

### 3.7. Spatial Cytokine Networks

Cytokines act over restricted distances, so different regions of one specimen may show distinct inflammatory programmes. ST can map ligand and receptor transcripts and the proximity of candidate sender and receiver cells, making it valuable for hypothesis generation. Ligand-receptor tools infer compatibility from expression databases, abundance, and sometimes spatial proximity. Their output depends on cell annotation, detection thresholds, database content, and the background model. A high communication score is computational evidence, not proof of ligand secretion, receptor occupancy, or downstream signalling. A proposed interaction should be strengthened sequentially by in situ RNA or protein co-localisation, receptor or pathway activation, perturbation or blockade, reproducibility across patients, and association with clinical outcomes. Table 2 and Table 5 therefore label IL-33-ST2, CCL26-CCR3, SPP1-CD44, TGF-β-TGFBR, CXCL12-CXCR4, and TNF-TNFR by source, platform, evidence type, and validation method.

**Table 5:** Evidence-graded cell-cell communication axes in chronic rhinosinusitis niches.

Niche/Sender → Receiver	Axis	Source/Platform	Spatial Evidence	Protein/In Situ	Functional Validation	Evidence-Based Interpretation
Injured epithelium → ILC2/eosinophil	IL-33-ST2	Shaw et al., 2013 [61]; Baba et al., 2014 [66]; no direct CRS ST	Not directly demonstrated	Tissue ST2/IL-33 localisation	Ex vivo IL-33-responsive ILC cytokine production	Supported non-ST axis; spatial organisation and clinical utility remain unconfirmed
IL-13-responsive epithelium → eosinophil	CCL26-CCR3	Min et al., 2017 [17]; Li et al., 2019 [18]; no direct CRS ST	Not directly demonstrated	Tissue/secreted chemokine support	Epithelial-cell and chemotactic support	Plausible recruitment axis; do not label as a confirmed ST interaction
ALOX15^+^ macrophage → eosinophil/epithelium	CCL13-associated axis	Liu et al., 2024 [7]; digital ST	ROI localisation/association	IHC/IF	Co-culture, inhibition and blockade	One of the stronger CRS niche mechanisms, but the full in vivo sender-receiver circuit remains incomplete
CXCL12^+^ fibroblast → GZMK^+^ CD8 T cell	CXCL12-CXCR4	Guo et al., 2024 [8]; Visium + Visium HD	Spatial adjacency and transcript co-expression	Multiplex imaging	GZMK→fibroblast response tested	Computationally predicted recruitment/retention edge
SPP1^+^ macrophage → fibroblast/type 2 cell	SPP1-CD44	Zhou et al., 2026 [19]; no ST	None	Human RT-qPCR/IHC/IF	Murine metabolic intervention; no pair-specific blockade	CellChat prediction supported by macrophage validation, not direct receptor-level causality
Stromal/immune source → epithelium/fibroblast	TGF-β-TGFBR	West et al., 2026 [10]; GeoMx	Pathway-level spatial change	RNA/protein panel and external literature	Directly not validated through ST analysis	Remodelling pathway is supported; specific pair and direction remain incompletely tested
Immune/mast-cell source → epithelium/fibroblast	TNF-TNFR	No direct CRS ST study identified	None	General/non-spatial inflammatory support	Not directly established for the proposed CRS niche	Extrapolated hypothesis requiring localisation and receptor-specific perturbation

### 3.8. Emerging Concept: The Sinonasal Immune Ecosystem

**Figure 2:**
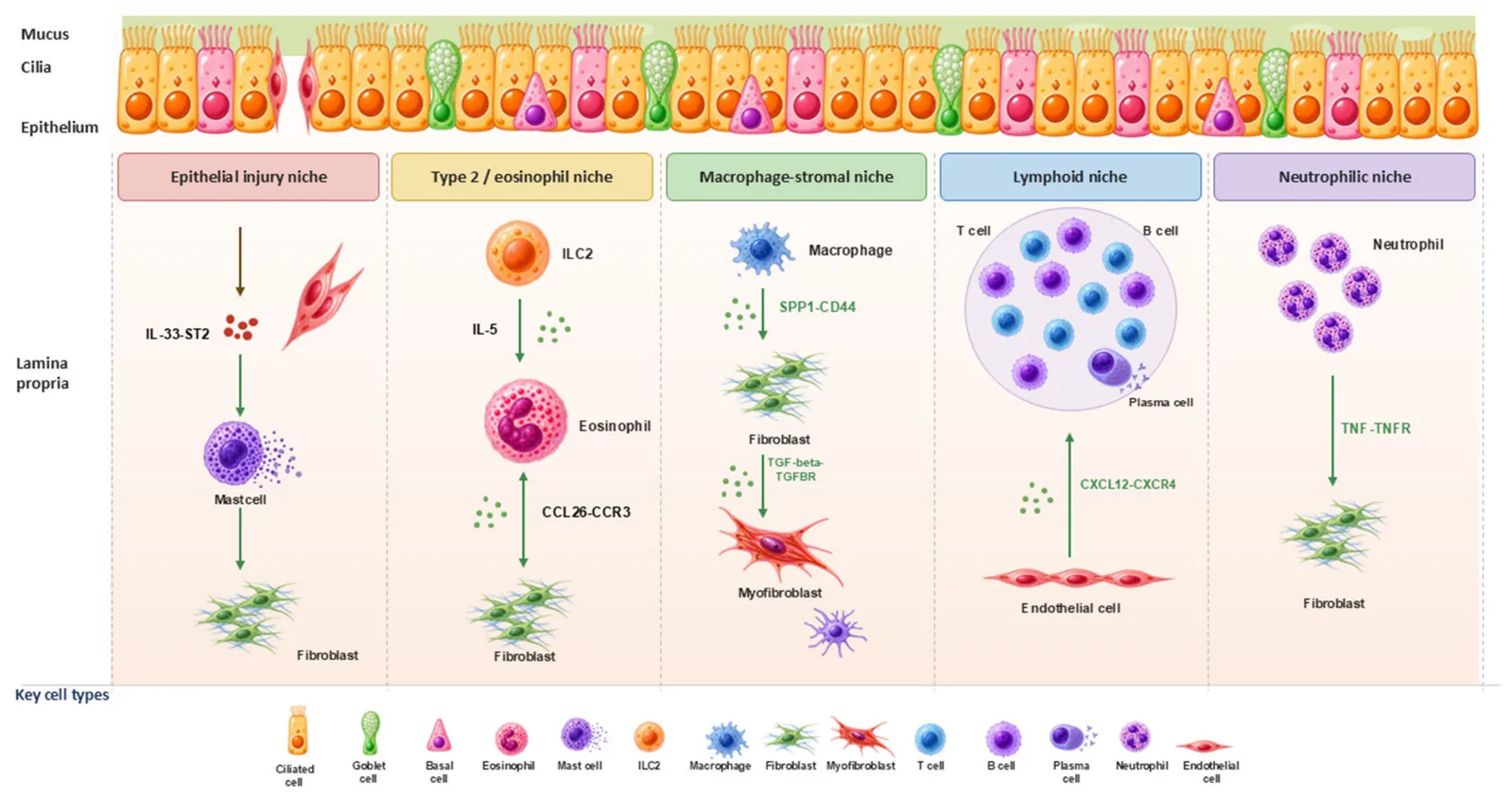
Conceptual schematic of spatially organised inflammatory and stromal niches in the airway mucosa. The diagram summarises five epithelial-lamina propria microenvironmental niches that may contribute to inflammation and tissue remodelling. Arrows denote possible or suspected associations. IL-33-ST2, IL-5, and CCL26-CCR3 have varying degrees of non-spatial CRS support, whereas the macrophage-stromal SPP1-CD44 and TGF-beta-TGFBR links are candidate interactions. Direct CRS spatial evidence, receptor-level validation, and causal direction have not been established for the latter two pairs. CXCL12-CXCR4 is shown as a predicted recruitment/retention circuit within the lymphoid niche.

CRS can be interpreted as a spatially organised tissue ecosystem in which epithelial, immune, stromal, and vascular compartments interact within specialised niches. This framework explains why bulk averages and phenotype labels may obscure clinically relevant heterogeneity. Each niche has a molecular signature, cellular composition, and anatomical context that can generate a mechanistic hypothesis. Translation requires testing whether disrupting the nominated circuit changes tissue function and whether a reproducible signature predicts an outcome in independent patients. Figure 2 distinguishes interactions supported beyond transcript co-expression from computationally predicted or proposed interactions.

## 4. Spatial Regulation of Epithelial Remodeling, Stromal Plasticity, and Tissue Architecture in Chronic Rhinosinusitis

Structural remodelling in CRS includes epithelial injury, altered differentiation, barrier failure, ECM deposition, angiogenesis, and hypoxia. Spatial profiling can localise these programmes, but many mechanistic conclusions continue to rely on scRNA-seq, histology, protein assays, and cell or tissue experiments. This section therefore states explicitly when evidence is spatial, orthogonal, or extrapolated. The central model is reciprocal rather than one-directional: inflammatory cells influence epithelial and stromal states, while damaged epithelium and activated fibroblasts shape immune recruitment. Spatial co-occurrence identifies candidate remodelling niches; causality requires lineage, perturbation, or longitudinal evidence.

### 4.1. Epithelial Injury and Inflammatory Memory

Epithelial erosion and type 2 cytokine exposure are spatially associated with abnormal secretory and repair programmes. Separate scRNA-seq and culture experiments show that basal progenitor cells from nasal polyps can retain an IL-4, IL-13-associated inflammatory memory after removal from tissue [11]. 

### 4.2. Basal Progenitor Plasticity and Differentiation

Multi-scale CRS atlases identify altered basal-cell and tuft-cell trajectories in relation to immune infiltration [9]. Trajectory methods infer likely state transitions from cross-sectional data; they do not directly observe lineage progression. Spatial proximity can constrain these inferences by showing where progenitor and differentiated states occur. The strongest conclusion is that epithelial differentiation is regionally altered and associated with local inflammatory programmes. 

### 4.3. Secretory Remodelling and Mucus Programmes

IL-13-responsive, goblet-cell, and mucin programmes can be concentrated in selected epithelial regions rather than distributed uniformly. Histology and transcriptomics support MUC5AC-rich secretory remodelling, while spatial studies localise associated epithelial states. These observations support a regional response to type 2 inflammation.

### 4.4. Loss of Ciliated Cells and Impaired Mucociliary Clearance

Mucociliary clearance is a principal innate defence of the upper airway. CRS tissue shows regional loss of mature ciliated-cell programmes, including FOXJ1- and axoneme-associated genes, adjacent to inflamed or remodelled epithelium. Spatial maps can distinguish preserved ciliated regions from nearby epithelial domains with reduced ciliogenesis. West et al. observed treatment-associated changes in epithelial ciliary programmes during mepolizumab therapy [10], providing longitudinal spatial association rather than proof that IL-5 blockade directly repairs cilia.

### 4.5. Epithelial Barrier Dysfunction

Barrier dysfunction is marked by regional alteration of tight-junction and epithelial-repair genes. IL33, TSLP, and IL25 are plausible local alarmins, but transcript co-expression with nearby immune cells does not establish cytokine release or receptor activation. A reciprocal model-barrier failure permitting exposure while inflammation further impairs repair-is supported by convergent tissue and experimental evidence. 

### 4.6. Fibroblast Diversity and Stromal Remodeling

Fibroblast heterogeneity is supported by scRNA-seq and spatial mapping, with inflammatory, matrix-producing, and activated, myofibroblast-like states occupying different stromal regions. Candidate programmes include COL1A1/COL3A1, POSTN, FN1, TNC, SPARC, THY1, and ACTA2. Guo et al. spatially mapped GZMK-positive CD8 T-cell-fibroblast neighbourhoods and demonstrated fibroblast transcriptional responses to recombinant GZMK [8]. CXCL12-CXCR4 was inferred from transcript expression and proximity; the pair itself remains predictive unless receptor-specific blockade confirms signalling. Fibroblast state labels and causal functions should therefore be separated. Spatial clustering demonstrates localisation, while matrix production, chemotaxis, and contractility require protein and functional assays. Cross-platform and cross-patient replication is particularly important because segmentation and deconvolution can alter fibroblast boundaries and abundance.

### 4.7. Periostin as a Spatial Marker of Tissue Remodelling

Periostin (POSTN) is a well-studied protein marker of eosinophilic/type 2 CRS and localises to ECM and thickened basement-membrane regions. Tissue abundance associates with eosinophilia and remodelling, while nasal-polyp-derived fibroblast experiments link periostin with VEGF, CCL5, and eotaxin-2 regulation [39,40]. These data provide protein and functional support; periostin is not yet a standardised spatial predictor of biologic response, and validated cut-offs are lacking. Cohort and meta-analytic studies also support periostin as an eosinophilic CRS severity/recurrence biomarker candidate, although thresholds and clinical deployment remain unsettled [41,42].

### 4.8. Angiogenesis and Endothelial Remodelling

Histology and protein studies show angiogenesis and VEGF expression in nasal polyps, including endothelial, glandular, and fibroblast compartments [39,43,44]. ST can map vascular-associated transcripts, but vessel permeability and leukocyte trafficking require functional or imaging confirmation. Hypoxia-associated HIF-1α signalling has experimental links to epithelial-mesenchymal transition, tight-junction disruption, neutrophilic inflammation, and glucocorticoid resistance [45,46,47]. Thus, VEGFA, ANGPT2, CXCL12, and SELE are candidate spatial markers of activated vascular niches.

### 4.9. Neuroimmune Organisation

Neuroimmune communication has not yet been comprehensively mapped by human CRS ST. Sensory and autonomic fibres and neuropeptides such as substance P, calcitonin gene-related peptide (CGRP), and vasoactive intestinal peptide (VIP) have non-spatial tissue evidence in sinonasal disease [67]. Additional biochemical and salivary studies support altered substance P metabolism and detectable CGRP/VIP in rhinosinusitis but do not establish spatial cellular circuits [68,69]. 

## 5. Clinical Translation of Spatial Transcriptomics: Towards Precision Medicine in Chronic Rhinosinusitis

**Figure 3:**
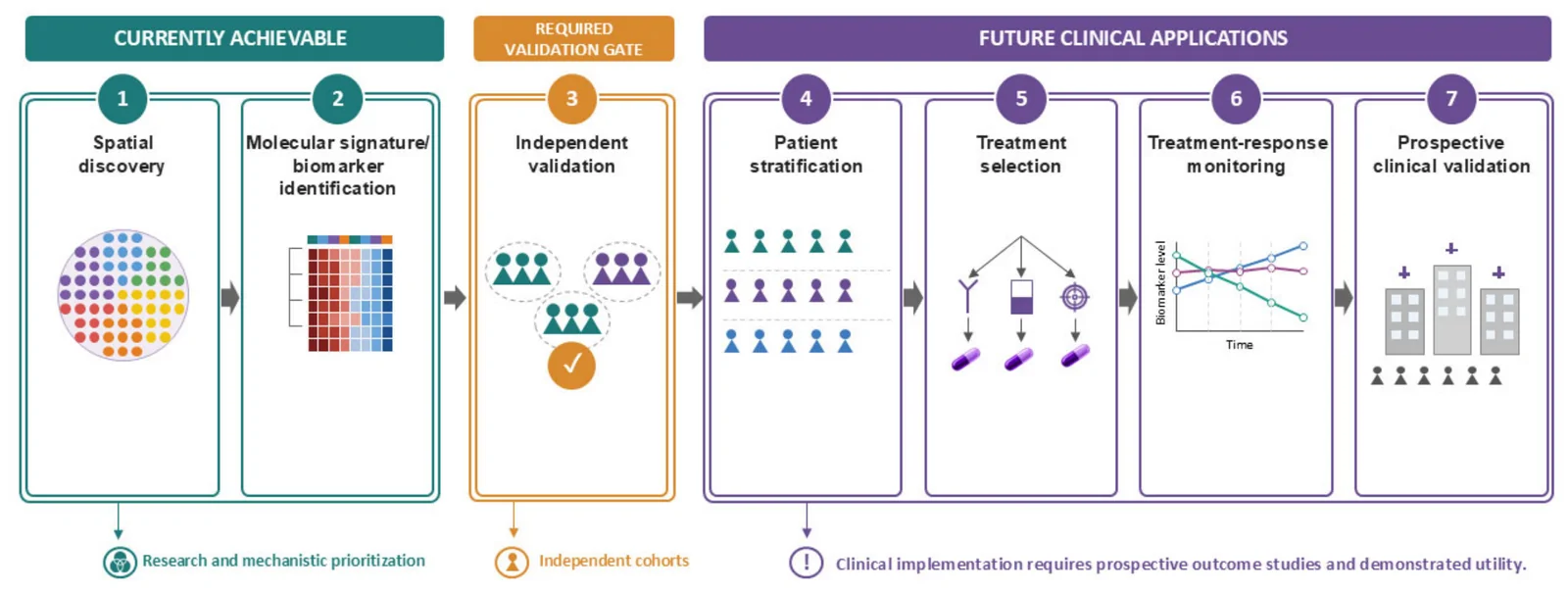
Precision-medicine framework for CRS. Spatial discovery and biomarker nomination are current research activities; patient stratification and treatment selection remain future applications that require analytical replication, independent validation, prospective outcome studies, and demonstrated clinical utility.

ST can connect tissue architecture with molecular endotypes, biomarker discovery, and treatment-response biology, but routine precision treatment is not yet achievable from a spatial assay alone. The current evidence supports discovery, localisation, and mechanistic prioritisation. Independent validation, assay standardisation, prespecified cut-offs, reproducibility, and prospective demonstration of clinical utility are required before patient stratification or biologic selection can be implemented. Figure 3 separates current research capability from validation gates and future clinical use.

### 5.1. From Histopathology to Spatial Molecular Pathology

Routine haematoxylin and eosin (H&E) sections show eosinophilia, fibrosis, epithelial damage, and glandular change but not all underlying molecular programmes. HE2Signature is a demonstrated proof of concept rather than a routine molecular surrogate: it used 70 transcriptome-paired nasal-polyp whole-slide images for model development, 30 for internal validation, and 224 patients from four centres for external testing. Internal endotype area under the receiver-operating-characteristic curve (AUC) values were 0.833, 0.903, and 0.935 for type 1, type 2, and type 3, respectively; in the external cohort, the image-predicted FCER2+CST1 combination identified type 2 disease with AUC 0.716. Twenty-eight of 33 predicted genes correlated positively with measured expression, and selected spatial predictions were compared with immunohistochemistry [70].

### 5.2. Spatial Biomarkers for Patient Stratification

A clinically useful spatial biomarker should be reproducible across tissue sites and patients and analytically measurable in a locked assay and should add predictive value beyond phenotype, histology, blood biomarkers, and comorbidities. Composite niches may be biologically richer than a single gene, but greater complexity increases sensitivity to sampling, segmentation, and model choice. Candidate type 2 features include IL-4/IL-13-responsive epithelium, eosinophils, mast cells, tuft-like cells, altered basal cells, and macrophage chemokines. Remodelling candidates include fibroblast/ECM and TGF-β-associated programmes; type 1/type 3 candidates include interferon, IL-17, antimicrobial, and neutrophil-associated programmes. 

### 5.3. Predicting Response to Biologic Therapy

Dupilumab, mepolizumab, benralizumab, and omalizumab target clinically validated type 2 pathways in appropriate CRSwNP populations, but their efficacy does not validate a spatial biomarker for drug choice. A baseline map may reveal IL-13-responsive epithelium, IL-5-associated eosinophilic regions or local IgE/plasma-cell architecture, yet these are hypotheses for prospective prediction. Spatial selection must outperform current eligibility criteria and be tested against clinical outcomes, not inferred from target presence. Table 4 distinguishes validated drug efficacy from unvalidated spatial treatment matching.

### 5.4. Monitoring Therapeutic Response

The strongest early translational example is longitudinal rather than predictive. West et al. analysed sinonasal biopsies from 20 patients before and after 16 and 24 weeks of mepolizumab, profiling 80 proteins, 1,833 stromal mRNA targets, and 18,815 epithelial transcripts. Region-specific changes involved immune pathways, TGF-β-associated processes, keratinisation, ciliogenesis, and epithelial-barrier biology [10]. This study shows that spatial assays can reveal tissue changes beyond blood eosinophil counts, but it does not establish a validated response-monitoring test or a baseline selector. 

### 5.5. Integration with Spatial Multi-Omics

RNA abundance is not equivalent to protein abundance or pathway activity. Integrating ST with multiplex protein imaging, metabolomics, epigenomics, and histology can strengthen localisation and mechanism, provided that differences in sensitivity, resolution, and dynamic range are explicitly modelled. Multimodal studies require robust registration and must report whether signals come from the same section, adjacent sections, or separately sampled tissue. Cross-modality concordance should be assessed at patient and region levels. 

### 5.6. Artificial Intelligence (AI) and Digital Pathology

AI can segment structures and predict molecular features from morphology, but demonstrated performance should not be conflated with clinical readiness. HE2Signature reported patient-separated internal and multicentre external evaluation, with strong internal endotype AUCs but more modest external type 2 discrimination (AUC 0.716) [70]. Generalisability remains vulnerable to staining, scanner, centre, tissue-processing, and population shifts. Future studies should report calibration, confidence intervals, failure cases, and subgroup performance; use fully external, patient-level test sets; compare against clinical baselines; and evaluate prospective utility before image-derived predictions influence treatment [71,72].

### 5.7. Current Limitations

Study design and sampling are the first constraints. Published CRS spatial cohorts are generally small relative to disease heterogeneity, and a single polyp, biopsy, or tissue site may not represent the molecular profile of the whole patient. CRSwNP, CRSsNP, eCRSwNP, and type 1, type 2, and type 3 programmes require consistent definitions. Anatomical site, prior treatment, surgery, comorbidity, ancestry, and disease severity introduce patient-to-patient variability. Multiple sections, ROIs, spots, or cells from one patient improve coverage but do not create independent biological replicates. Technical and analytical constraints include spot mixing, finite molecular sensitivity, spatial autocorrelation, multiple testing, segmentation error, reference-dataset dependency, deconvolution uncertainty, batch effects, and cross-platform normalisation. FFPE enables retrospective cohorts but may reduce RNA quality; fresh-frozen tissue can improve discovery but is harder to standardise clinically. Platform differences in targeted versus unbiased detection, tissue area, and dynamic range complicate replication. Analyses should use patient-level or hierarchical inference, preserve spatial structure in null models, report uncertainty and exclusions, and validate findings with in situ protein/RNA and perturbation assays.

### 5.8. Clinical Feasibility Turnaround and Implementation

Current ST platforms remain research workflows rather than routine diagnostic tests for CRS. Total cost and turnaround depend on platform, tissue preparation, probe or library chemistry, sequencing or imaging capacity, and data analysis; fixed estimates therefore cannot yet be generalised across institutions. Workflows commonly require batching and, in many settings, centralised or off-site processing, so they do not currently match the turnaround expected for routine surgical pathology. Before implementation, prospective studies should compare a spatial assay’s incremental clinical value and resource use with established histology, tissue eosinophil quantification, and blood or serum biomarkers. A practical staged pathway would begin with centralised discovery or reference laboratories and proceed to locked, smaller targeted panels only after analytical and clinical validation [24].

Moreover, spatial molecular pathology also requires coordinated infrastructure spanning the treating clinician and surgeon, pathologist and histotechnologist, molecular laboratory, bioinformatician, biostatistician, and clinical-trial team. For a clinical test, preprocessing, segmentation, deconvolution, and predictive-model thresholds must be locked before validation; performance needs to be evaluated with patient-level external datasets, calibration, subgroup analyses, and prospective clinical utility rather than inferred from technical accuracy alone [71,72].

### 5.9. Future Perspectives

The next priority is not simply to generate more maps, but to create comparable, patient-level maps across healthy mucosa, CRSsNP, CRSwNP, eCRSwNP, type 1, type 2, type 3 and mixed endotypes, relevant comorbidities, and diverse populations. Discovery cohorts should be followed by locked targeted panels, independent validation and longitudinal studies of biologic response, recurrence, and postoperative outcomes. 

## 6. Conclusions

Spatial transcriptomics adds a crucial ‘where’ to the molecular ‘what’ of CRS. Direct studies show that epithelial, immune, and stromal programmes are compartmentalised and can change during therapy, while integrated single-cell, protein, and functional evidence helps interpret those maps. The field’s unique opportunity is to connect spatial niche to molecular signature to plausible mechanism to phenotype/endotype to clinical hypothesis. Its central caution is equally important: co-localisation and ligand-receptor inference do not establish causality, and a drug’s efficacy does not validate a spatial selection biomarker. Pseudoreplication—treating spots, bins, ROIs, or cells from one patient as independent biological replicates—remains a principal barrier to reliable inference and should be prevented through patient-level or hierarchical study designs. With patient-level replication, orthogonal perturbation, independent multicentre cohorts, standardised assays, and prospective clinical utility studies, ST could mature from a discovery technology into a credible component of CRS phenotyping, biomarker development, and treatment monitoring.

## Figures and Tables

**Table 1:** Direct CRS spatial transcriptomic studies: population, platform, principal spatial finding, and key caveat. Detailed integrated methods, validation approaches, pathways, and limitations are provided in Supplementary Tables S1 and S2.

Study/Year/Source	CRS Population and Specimen	Platform/Resolution	Principal Spatial Finding	Key Caveat
Liu et al., 2024 [7]	eCRSwNP vs. neCRSwNP; eCRSwNP 46 and neCRSwNP 31; nasal-polyp epithelium/subepithelium	Digital spatial transcriptomics; ROI-level	ALOX15-positive macrophage programme localised with eosinophilic epithelial remodelling	Cross-sectional; ROI-level mixing; the complete in vivo circuit remains unproven.
Guo et al., 2024 [8]	CRSwNP vs. control inferior turbinate; ST: 3 polyp/2 control, plus HD 6 polyp/4 control	Visium 55-micrometre spots; Visium HD 2 x 2-micrometre bins	GZMK-positive CD8 T cells localise near CXCL12-positive fibroblasts; GZMK induces a fibroblast response	Small patient-level ST cohort; inferred interactions remain predictive.
Liao et al., 2025 [9]	CRSwNP, CRSsNP, and healthy tissue; discovery 6/4/5; GeoMx validation 61/45/7	GeoMx DSP; morphology-selected ROIs	Macrophage/eosinophil recruitment, altered T-cell programmes, mast-cell accumulation, and basal/tuft remodelling	ROI-selection bias; cross-sectional design; computational interactions do not prove causality.
West et al., 2026 [10]	eCRSwNP receiving mepolizumab; 20 paired biopsies at baseline and weeks 16 and 24	GeoMx DSP; ROI-level RNA and protein	Spatially distinct immune, stromal, and epithelial changes during IL-5 blockade	Small non-randomised cohort; no validated predictive threshold; direct versus indirect effects unresolved.
Cui et al., 2026 [12]	eCRSwNP/CRSwNP vs. control; discovery CRSwNP 30 and control 10; independent validation cohort	GeoMx DSP; ROI-level	Subepithelial ALOX15-positive macrophage-CCL13 programme associated with eosinophil recruitment	Recent single-centre study; ex-vivo pharmacology is not prospective clinical validation.

**Table 2:** Evidence audit of representative ligand-receptor axes discussed in chronic rhinosinusitis.

Ligand-Receptor Axis	Proposed Sender → Receiver	Source Study/CRS Context	Evidence	Supported Interpretation	Residual Uncertainty
CCL26-CCR3	IL-13-responsive epithelium to eosinophil	Min et al., 2017 [17]; Li et al., 2019 [18]	Tissue RNA/protein and epithelial-cell experimental support; chemotactic biology	Plausible eosinophil-recruitment axis	Spatial sender-receiver pairing and clinical predictive value are unvalidated
SPP1-CD44	SPP1^+^ macrophage to fibroblast/type 2 immune cell	Zhou et al., 2026 [19]	No direct CRS ST evidence; CellChat prediction; human RT-qPCR/IHC/IF; murine metabolic intervention	Macrophage state supported; SPP1-CD44 receptor-level signalling remains unproven	Specific SPP1-CD44 signalling in human CRS was not spatially or receptor-blockade validated
TGF-β-TGFBR	Stromal/immune source to epithelium/fibroblast	West et al., 2026 [10]; supporting non-spatial CRS literature	Spatial pathway-level association during therapy; protein/experimental literature	TGF-β-associated stromal and epithelial remodelling is plausible	A specific ligand-receptor event and causal direction were not shown by the spatial study
CXCL12-CXCR4	CXCL12^+^ fibroblast to GZMK^+^ CD8 T cell	Guo et al., 2024 [8]	Transcript co-expression, spatial adjacency and CellChat prediction; IF; downstream GZMK perturbation	Candidate recruitment/retention circuit in a T-cell-fibroblast niche	CXCL12/CXCR4-specific blockade was not reported

**Table 3:** Airway-mucosal quick-reference comparison of spatial transcriptomic platforms: resolution, capture area/throughput, sensitivity, and specimen considerations.

Platform	Spatial Resolution	Capture Area/Throughput	Sensitivity and Analytical Constraints	Airway-Mucosal Use and Specimen Considerations
Visium/Visium HD	55-micrometre spots/2 × 2-micrometre bins	6.5 or 11 mm capture areas; multiple reactions per slide	Broad discovery sensitivity; spot mixing or segmentation affects cell assignment; sequencing and deconvolution are required.	Frozen and FFPE workflows; tissue-wide epithelial-stromal discovery. Record section orientation, tissue coverage, and epithelial integrity.
GeoMx DSP	ROI/AOI level; segmentation dependent	Up to about 96 ROIs per viewing area; morphology-selected regions	ROI aggregation improves sensitivity but masks within-ROI cellular heterogeneity; selection bias remains.	Strong FFPE compatibility; well suited to archived specimens and paired biopsies with pathologist-defined epithelial, stromal, immune, or vascular ROIs.
MERFISH	Single-molecule; subcellular	Centimetre-scale areas; acquisition depends on panel and imaging system	High molecular specificity but targeted; autofluorescence, probe performance, registration, and segmentation matter.	High-resolution validation in fixed tissue; pilot optimisation should assess mucus/autofluorescence and segmentation in airway sections.
seqFISH+	Single-molecule; subcellular	Research-scale fields/sections; many imaging cycles	High multiplexity but demanding chemistry, imaging, decoding, registration, and error control.	Mechanistic research and dense targeted mapping; not yet a routine airway-mucosal workflow.
Xenium In Situ	Subcellular transcripts; segmented cells	Up to two slides per run; assay-dependent areas and less-than-one-week workflow	High localisation but panel and segmentation limited; targeted design can miss unanticipated biology.	Candidate localisation and validation in FFPE or frozen airway tissue after panel and morphology optimisation.
CosMx SMI	Single-cell/subcellular	Large fields; up to more than one million cells, assay dependent	High-plex RNA/protein context but autofluorescence, crowding, panel effects, and large image data require care.	RNA-protein validation and neighbourhood analysis in FFPE or frozen airway tissue; requires substantial image-data infrastructure.
Stereo-seq	About 220-nm spots/500-nm pitch	Chips from millimetres to centimetres	High spatial density; capture and sequencing depth vary; compute, image alignment, binning, and segmentation are intensive.	Large-area, high-resolution discovery; currently specialised rather than routine for airway mucosa.
Seq-Scope	About 0.5-0.7-micrometre array coordinates	Approximately 7 × 7 mm or larger custom arrays	Very high spatial density but custom array production, coordinate decoding, segmentation, and data volume are demanding.	Specialised epithelial or subcellular discovery; primarily a fresh-frozen research workflow.

**Table 4:** Candidate spatial endotypes, therapeutic relevance, and current validation status.

Candidate Spatial Endotype/Phenotype	Candidate Tissue Signature	Therapeutic Relevance	Evidence/Validation	Current Status	Critical Limitation
Type 2 CRSwNP/eCRSwNP	IL-4/IL-13-responsive epithelium; eosinophils; POSTN; mast/ILC2 programmes	Dupilumab (IL-4Rα)	Drug efficacy clinically validated in CRSwNP [3]; spatial niche supported by CRS ST and orthogonal studies [7,9]	Biologic is established; spatial selection is investigational	No prospective evidence that the spatial signature selects dupilumab better than current criteria
Eosinophilic type 2 CRSwNP	IL-5-associated eosinophil niches; CLC/EPX/PRG2	Mepolizumab (IL-5); benralizumab (IL-5Rα)	Clinical efficacy established in selected CRSwNP [3]; GeoMx longitudinal tissue changes shown for mepolizumab [10]	Monitoring is emerging; selection unvalidated	Treatment-associated spatial change is not a predictive baseline biomarker
IgE/plasma-cell-rich CRSwNP	Local IgE, plasma cells and TLS architecture	Omalizumab (IgE)	Omalizumab efficacy clinically validated [3]; local immune architecture supported mainly by histology/protein/non-ST transcriptomics [26,27,28,29,30]	Spatial matching is hypothetical	No validated tissue-spatial threshold predicts response
Type 1/type 3/neutrophilic CRS	Interferon/STAT1, IL-17 and S100A8/A9/LCN2 programmes	Potential innate or IL-17-pathway targeting	Biological support from non-spatial and single-cell studies [31,32,33,34,35,36,37,38]; limited direct CRS ST	No established spatially selected biologic	Heterogeneous, overlapping programmes and insufficient prospective trials
Fibrotic/remodelling-dominant CRS	TGF-β, ECM, POSTN, activated fibroblasts, hypoxia	Anti-remodelling strategies; pathway modulation	Spatial pathway association plus protein/functional support [10,39,40,41,42,43,44,45,46,47]	Mechanistic research	No approved spatially guided therapy; causal drivers and safety require testing
Mixed endotype	Coexisting type 2, innate and remodelling niches	Combination or sequential strategies	Spatial coexistence is plausible and increasingly observed [9,31,32,33,34,35,36,37,38]	Future possibility	Possible sampling error, assay complexity and lack of prospective utility data

## Data Availability

No new data were created or analysed in this study.

## Supplementary Materials

The following supporting information can be downloaded at: https://www.mdpi.com/article/10.3390/ijms27219510/s1.

## Abbreviations

**2-DG**	2-Deoxy-D-glucose
**ACTA2**	Actin alpha 2, smooth muscle
**AI**	Artificial intelligence
**ALOX15**	Arachidonate 15-lipoxygenase
**ANGPT2**	Angiopoietin 2
**AOI**	Area of interest
**AUC**	Area under the receiver operating characteristic curve
**BCR**	B-cell receptor
**CCL5**	C–C motif chemokine ligand 5
**CCL13**	C–C motif chemokine ligand 13
**CCL18**	C–C motif chemokine ligand 18
**CCL26**	C–C motif chemokine ligand 26
**CCR3**	C–C motif chemokine receptor 3
**CD8**	Cluster of differentiation 8
**CD44**	Cluster of differentiation 44
**CGRP**	Calcitonin gene-related peptide
**CLC**	Charcot–Leyden crystal galectin
**COL1A1**	Collagen type I alpha 1 chain
**COL3A1**	Collagen type III alpha 1 chain
**CRS**	Chronic rhinosinusitis
**CRSsNP**	Chronic rhinosinusitis without nasal polyps
**CRSwNP**	Chronic rhinosinusitis with nasal polyps
**CST1**	Cystatin SN
**CXCL12**	C–X–C motif chemokine ligand 12
**CXCL13**	C–X–C motif chemokine ligand 13
**CXCR4**	C–X–C motif chemokine receptor 4
**DNA**	Deoxyribonucleic acid
**DOI**	Digital object identifier
**DSP**	Digital Spatial Profiling
**ECM**	Extracellular matrix
**eCRSwNP**	Eosinophilic chronic rhinosinusitis with nasal polyps
**EMT**	Epithelial-to-mesenchymal transition
**EPX**	Eosinophil peroxidase
**ERK**	Extracellular signal-regulated kinase
**FCER2**	Fc epsilon receptor II
**FF**	Fresh-frozen
**FFPE**	Formalin-fixed, paraffin-embedded
**FN1**	Fibronectin 1
**FOXJ1**	Forkhead box J1
**GZMK**	Granzyme K
**HD**	High-definition
**HIF-1α**	Hypoxia-inducible factor 1 alpha
**IF**	Immunofluorescence
**IFN**	Interferon
**IgE**	Immunoglobulin E
**IHC**	Immunohistochemistry
**IL**	Interleukin
**IL-1β**	Interleukin 1 beta
**IL-4**	Interleukin 4
**IL-5**	Interleukin 5
**IL-5Rα**	Interleukin 5 receptor alpha
**IL-13**	Interleukin 13
**IL-17**	Interleukin 17
**IL-17A**	Interleukin 17A
**IL-25**	Interleukin 25
**IL-33**	Interleukin 33
**IL-4Rα**	Interleukin 4 receptor alpha
**ILC**	Innate lymphoid cell
**ILC2**	Group 2 innate lymphoid cell
**LCN2**	Lipocalin 2
**M1/M2**	Classically activated/alternatively activated macrophage states
**MERFISH**	Multiplexed error-robust fluorescence in situ hybridisation
**MERTK**	MER proto-oncogene tyrosine kinase
**MUC5AC**	Mucin 5AC
**MUC5B**	Mucin 5B
**neCRSwNP**	Non-eosinophilic chronic rhinosinusitis with nasal polyps
**PMID**	PubMed identifier
**POSTN**	Periostin
**PRG2**	Proteoglycan 2
**PTPN2**	Protein tyrosine phosphatase non-receptor type 2
**QC**	Quality control
**RNA**	Ribonucleic acid
**RNA-seq**	RNA sequencing
**RNASE2**	Ribonuclease A family member 2
**ROI**	Region of interest
**RT-qPCR**	Reverse-transcription quantitative polymerase chain reaction
**S100A8**	S100 calcium-binding protein A8
**S100A9**	S100 calcium-binding protein A9
**scRNA-seq**	Single-cell RNA sequencing
**SELE**	Selectin E
**seqFISH+**	Sequential fluorescence in situ hybridisation plus
**SMI**	Spatial Molecular Imaging
**SPARC**	Secreted protein acidic and cysteine rich
**SPP1**	Secreted phosphoprotein 1
**ST**	Spatial transcriptomics
**ST2**	Suppression of tumorigenicity 2, also known as interleukin-1 receptor-like 1
**STAT1**	Signal transducer and activator of transcription 1
**TCR**	T-cell receptor
**TGF-β**	Transforming growth factor beta
**TGFBR**	Transforming growth factor beta receptor
**THP-1**	Human monocytic leukaemia cell line THP-1
**THY1**	Thy-1 cell-surface antigen
**TLS**	Tertiary lymphoid structure
**TNC**	Tenascin C
**TNF**	Tumour necrosis factor
**TNFR**	Tumour necrosis factor receptor
**TSLP**	Thymic stromal lymphopoietin
**VEGF**	Vascular endothelial growth factor
**VEGFA**	Vascular endothelial growth factor A
**VIP**	Vasoactive intestinal peptide
