# Supplementary material for: Spatial Transcriptomics in Chronic Rhinosinusitis: Deciphering Tissue Architecture, Immune Niches, and Precision Therapeutics
Source: Int J Mol Sci. 2026 Oct 24;27(21):9510. doi: 10.3390/ijms27219510 (PMC13762752; doi:10.3390/ijms27219510)
Supplement: Supplementary file 1 [file ijms-27-09510-s001.zip › ijms-4605860-supplementary.pdf]

Supplementary Table S1: Detailed methods validation and pathway information for direct CRS spatial transcriptomic studies

Study	Integrated methods	Validation approach	Key pathways	Detailed limitations
Liu et al., 2024 [7]	scRNA-seq; histology; pathway and communication analyses	IHC/IF; primary epithelial-THP-1 co-culture; ALOX15 inhibition / CCL13 blockade	ALOX15; CCL13; type 2 inflammation; epithelial repair	Cross-sectional; ROI-level mixing; functional model does not prove the complete in-vivo circuit.
Guo et al., 2024 [8]	scRNA-seq; TCR/BCR sequencing; public scRNA integration; CellChat	Multiplex IHC/IF; spatial co-localisation; primary polyp fibroblasts plus recombinant GZMK; bulk RNA-seq	GZMK; CXCL12/CXCR4; fibroblast activation	Small patient-level ST cohort; interaction inference is predictive; HD bins still require segmentation or aggregation.
Liao et al., 2025 [9]	scRNA-seq; trajectory and cell-cell communication analyses	Independent spatial cohort (n=113); histological/morphology anchoring	CCL13/CCL18; type 2 and mixed inflammation; epithelial differentiation	ROI-selection bias; cross-sectional design; computational interactions do not establish causality.
West et al., 2026 [10]	80 proteins; 1,833 stromal mRNAs; 18,815 epithelial transcripts	Within-patient longitudinal comparison; RNA-protein integration	Eosinophil biology; TGF-beta/EMT; barrier; keratinisation; ciliogenesis	Small non-randomised tissue cohort; no validated predictive threshold; direct versus indirect treatment effects unresolved.
Cui et al., 2026 [12]	Bulk and single-cell transcriptomic integration; pathway analysis	Human sinonasal explants; chemotaxis/migration assays; IL-4Ralpha pathway blockade; tissue validation	ALOX15; CCL13; IL-4/IL-13-associated macrophage activation	Recent single-centre study; cohort details differ by analytic subset; ex-vivo pharmacology is not prospective clinical validation.

Supplementary Table S2: Study-specific eCRSwNP definitions and tissue-eosinophil thresholds in direct CRS spatial studies

Study	Study-specific eCRSwNP definition / tissue-eosinophil threshold
Liu et al., 2024 [7]	eCRSwNP: >10% tissue eosinophils among total infiltrating cells.
West et al., 2026 [10]	eCRSwNP: >10 tissue eosinophils per high-power field.
Cui et al., 2026 [12]	The study reported analyses using tissue-eosinophil cut-offs of 10 and 30 eosinophils per high-power field.
